# Integrated transcriptomic and metabolomic analyses revealed the molecular mechanism of terpenoid formation for salicylic acid resistance in *Pulsatilla chinensis* callus

**DOI:** 10.3389/fpls.2022.1054317

**Published:** 2023-01-06

**Authors:** Yanjing Dong, Qian Qin, Guoyue Zhong, Zejing Mu, Yating Cai, Xiaoyun Wang, Huan Xie, Shouwen Zhang

**Affiliations:** ^1^ Research Center of Traditional Chinese Medicine Resources and Ethnic Minority Medicine, Jiangxi University of Chinese Medicine, Jiangxi, China; ^2^ Pharmacy school of Nanchang Medical College, Nanchang, China

**Keywords:** *Pulsatilla chinensis*, callus, salicylic acid, suspension culture, transcriptomics, metabolomics, terpenoid components

## Abstract

As a kind of traditional Chinese medicine, *Pulsatilla chinensis* (Bunge) Regel is well known for its anti-inflammation and anti-cancer activities, which are attributed to its active components including total saponins and monomers. To clarify the synthesis and metabolism mechanisms of class components in callus terpenes of *P. chinensis*, a certain concentration of salicylic acid (SA) hormone elicitor was added to the callus before being analysed by transcriptomic and metabolomic techniques. Results showed that the content of *Pulsatilla* saponin B4 in the callus suspension culture was significantly increased up to 1.99% with the addition of SA. Kyoto Encyclopedia of Genes and Genomes (KEGG) enrichment analysis showed that the differentially expressed genes were mainly enriched in 122 metabolic pathways, such as terpenoid metabolism-related pathways: terpenoid skeleton synthesis pathway, monoterpenoid biosynthesis pathways, diterpenoid biosynthesis pathways, and ubiquinone and other terpenoid-quinone biosynthesis pathways. A total of 31 differentially accumulated metabolites were obtained from four differential groups. Amongst 21 kinds of known chemical components in *P. chinensis*, deoxyloganic acid was the only monoterpenoid; the others are triterpenoids. In summary, this study found that SA elicitors can affect the metabolic changes of terpenoids in *P. chinensis* callus, which provided a basis for analysing the genetic regulation of terpenoid components of leucons.

## Introduction

Pasqueflower (*Pulsatilla chinensis* (Bge.) Regei) is a traditional Chinese medicine, belonging to the Ranunculaceae family. Pasqueflower features with the following aspects, such as its root is dry in nature, its taste is bitter and cold, and it can be absorbed by stomach and large intestine (Commission, 2020). At present, *P. chinensis* is mainly distributed in Liaoning, Jilin, Heilongjiang, Inner Mongolia, Shandong, Anhui, Shanxi, and Shaanxi Provinces (Yang and Liwei, 2012; Qian et al., 2017). It has various functions, including repairing heat injury, detoxification and soothing blood pressure; it is used for the symptoms of blood toxin and heat pain, vaginal itching, and discharge. The terpenoid and monomers in *P. chinensis* are widely adapted to clinical and veterinary treatment for their anti-cancer activity (Kang et al., 2019; Huihui Song et al., 2021). Pulsatilla saponins B3, BD, B4, B7, B9, B10, B11, A3, D, PSA, R13 and other components have been widely reported for their significant pharmacological activities (Xu et al., 2013; Liu, 2014; Liang, 2016; Guo et al., 2018; Yingying Luo et al., 2018; Kang et al., 2019; Yaru Cui et al., 2019; Zhang et al., 2021). Yingying Luo isolated and identified the total saponins of *P. chinensis* and the isolated monomers to study anti-tumor activity *in vitro*, and found that monomers of B3, B7, B9 and B11 could significantly inhibit tumor proliferation (Luo, 2014). Pulsatilla saponin R13 can inhibit metastatic activity of tumor by inhibiting G1/S phase transduction, inducing apoptosis, regulating cell energy metabolism and promoting cell autophagy (Liang, 2016). Pulsatilla saponin A3 acted on TLR4/NF- κ B/MAPK signal pathway to induce M1 macrophage polarization to inhibit breast cancer (Yin et al., 2021).As a natural compound-originated immunomodulatory agent, Pulsatilla saponin B4 is used to treat a variety of inflammatory diseases, such as ulcerative colitis (Li et al., 2020).

Cell suspension culture, callus culture, hairy root culture and other technologies have been widely used to large-scale cultivation and production of medicinal plant resources to obtain secondary metabolites of medicinal plants (Fan Zhantao et al., 2022). At present, the cell suspension culture technology of medicinal plants such as Taxus chinensis (Bai, 2020), Achyranthes bidentata (Ping Li et al., 2020), Cyclocarya paliurus (Zhao, 2020) and Scutellaria baicalensis (Wang, 2020) have been developed well. Hormone elicitors such as salicylic acid (SA) and methyl jasmonate (MeJA) are often used to regulate the production of secondary metabolites of medicinal plants (Xiaofeng Song et al., 2020; Xiaoxia Yan et al., 2020; Zhang, 2021; Ruixiang Lei et al., 2021; Ming Wang et al., 2021; Qiang Zhang et al., 2021; Li, 2021). The callus of Achyranthes bidentata and its biomass and secondary metabolite content were observed after adding certain concentrations of SA and MeJA. An addition of 1 mg/L SA could promote the accumulation of callus biomass and secondary metabolite content (Guo, 2016). When the callus of Gentiana macrophylla were cultured in suspension, the contents of total phenols, total flavonoids and other secondary metabolites changed significantly followed by addition of different concentrations of SA (Ruixiang Lei et al., 2021). In another research, SA and MeJA can promote the accumulation of alkaloids (Yongbo Duan et al., 2017).

Terpenoids are the main active components of Pulsatilla chinensis. Researches have showed that the skeleton biosynthesis of terpenoids mainly involves shikimic acid pathway, mevalonic acid (MVA) pathway, and methyl erythritol 4-phosphate (MEP) pathway (Ma, 2011; Li, 2022). Among them, squalene synthetase (SS), squalene epoxidase (SE), oxidized squalene cyclase (OSC), cytochrome P450 and glycosyltransferase (UGT) are key enzymes in these pathways (Yunsheng Zhao et al., 2009; Liao, 2010; Yuan Zhu et al., 2020).

The biosynthesis of secondary metabolites in medicinal plants is based on molecular genetics studies, the core of which is functional gene research. Under the regulation of functional genes, medicinal plants synthesize and accumulate secondary metabolites to produce active ingredients, or heterologously synthesize functional ingredients by microorganisms. With the development of high-throughput sequencing technology, multiomics studies have been gradually applied to the analysis of synthetic pathways of secondary metabolites in medicinal plants. Especially for medicinal plants without reference genomes, transcriptome and metabolome technologies are effective methods in analysing the synthesis pathways of secondary metabolites and to mine related functional genes.

Based on these advanced methods, mine terpenoid synthesis accumulation functional genes, and synthesis mechanism of *Pulsatilla* terpenoid metabolites could be analysed by transcriptome and metabolome technologies.

## Materials and methods

### Plant materials, culture conditions, and SA treatment

In this experiment, callus of 3th generation (3 months) were cultured as materials. It took about 18 days after the suspension culture in the middle exponential growth stage and about 30 days in the initial stage of platform for the callus. The following are the culture conditions of callus suspension: MS + 1.2 mg/L TDZ + 0.1 mg/L NAA + 2.0 mg/L VC + 20 g/L sucrose (Xiaozhen, 2020), medium volume 50 mL/bottle, inoculum volume 1 g/bottle, culture conditions 25 °C, 120 r/min shaking dark culture.

For the preparation of SA elicitor, 138.12 mg of SA was weighed and dissolved in 10 mL absolute ethanol. Then, it was sterilised by filtration. Next, 0 μmol/L, 100 μmol/L, 200 μmol/L, 300 μmol/L and 400 μmol/L SA elicitors were drawn into the callus liquid medium cultured on the 18th and 30th day, and samples were taken after co-cultivation for 2 days.

### Effects of SA on callus growth and accumulation of secondary metabolite saponin B4

**Figure 1:**
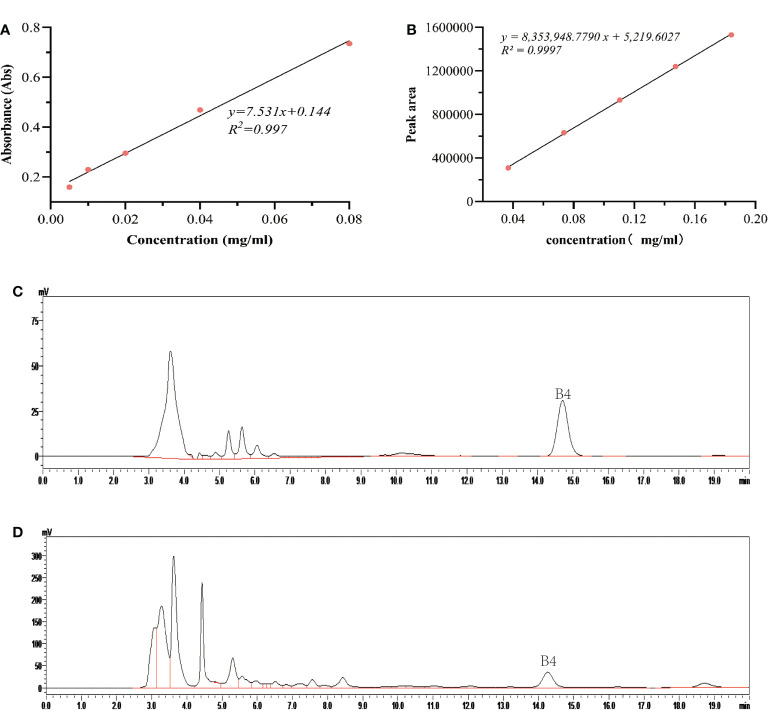
Effects of salicylic acid on carbon source consumption and saponin B4 accumulation in callus. **(A)** Standard curve of glucose; **(B)** Standard curve of Pulsatilla saponin B4; **(C)** High performance liquid chromatography (HPLC) of reference substance; **(D)** Sample HPLC.

The weight of fresh callus, conductivity of culture medium, pH value, sugar concentration, saponin B4 content, etc. were measured. The determination of sugar concentration was referred to the anthrone sulfate method (Zhu et al., 2018) in the culture solution, and the detection wavelength was 582 nm. Draw standard curve y=7.531x+0.144, R^2 =^ 0.997, and calculate sugar concentration in the culture medium (**Figure 1A**).

For determination of saponin B4 content, callus was taken out, dried at 50°C, ground into powder, and refrigerated for later use. Chromatographic conditions: chromatographic column, YMC-Pack ODS-A (4.6 × 250 mm, 5 µm); mobile phase: acetonitrile:water = 28:72, flow rate 0.8 mL/min, injection volume 20 µl, column temperature 30°C, detection wavelength 203 nm. For sample preparation, weigh about 0.1000 g of *P. chinensis* powder and add it to a 50 mL conical flask with stopper; add 10 mL of methanol and seal with parafilm tightly. The sample was extracted ultrasonically (power 150 W, frequency 40 kHz) for 40 min. Then, let it cool and make up the weight with pure methanol, shake well, filter, and spin dry the filtrate. Extract the sample with water-saturated n-butanol for three times (20 mL each time), combine with n-butanol solution, spin dry, add methanol to the residue to dissolve, dilute to a 10 mL volumetric flask, filter through a 0.22 μm microporous membrane, and set aside. To draw the standard curve, weigh an appropriate amount of *Pulsatilla officinalis* B4 reference substance, dissolve in methanol, and dilute in a 10 mL volumetric flask to prepare a stock solution with a concentration of 1.840 mg/mL. Accurately draw the reference substance stock solution of *Pulsatilla officinalis*, and dilute it to 0.0368, 0.0736, 0.1104, 0.1472, and 0.184 mg/mL reference substance solution in turn. The injection volume was 20 µl. The peak area of the chromatographic peak was measured, and the standard curve of *Pulsatilla* saponin B4 was drawn (**Figure 1B**): y = 8353948.7790 x + 5219.6027 (R² = 0.9997), and the linearity range was 0.768–3.680 μg. The HPLC chromatograms of the reference substance and sample were obtained (**Figure 1C, D**).

### RNA extraction, library construction, and transcriptome sequencing

The above concentrations of SA elicitors that can significantly increase the content of *Pulsatilla* saponin B4 were obtained through screening and co-cultured with *P. chinensis* callus for 0, 1, 2 and 3 days. Each treatment was repeated three times, with a total of 12 samples. The samples were washed 2–3 times with sterile water, snap-frozen in liquid nitrogen, and stored in -80°C refrigerator for transcriptome and metabolome sequencing research.

The RNA of samples was extracted using a versatile plant RNA extraction kit (DNase I). The concentration and purity of the extracted RNA were tested with a NanoDrop 2000 spectrophotometer. RNA integrity was detected by agarose gel electrophoresis, and RIN was measured with an Agilent 2100 bioanalyser. Total RNA of ≥1 µg, concentration of ≥35 ng/μl, D260/280 ≥1.8, OD260/230 ≥1.0, and RIN value ≥6.5 were required for single library construction. The cDNA library was constructed after the samples were qualified. The constructed library was sequenced on the Illumina NovaSeq 6000 platform.

The RNA purity, concentration, and integrity of samples were tested. The total amount meets the requirements of two standard database constructions, and subsequent experiments can be carried out (**Supplementary Table 1**). To obtain high-quality quality control data (clean data), the original sequencing data were quality-controlled to ensure the accuracy of subsequent analysis results. (**Supplementary Table 2**).

### *De novo* assembly and functional annotation

For transcriptome studies without reference genomes, after obtaining high-quality RNA-seq sequencing data, all quality-controlled sequencing reads need to be assembled *de novo* to generate contigs and singletons. The software used was Trinity (https://github.com/trinityrnaseq/trinityrnaseq/wiki) (Grabherr et al., 2011). After being spliced with Trinity, the assembly results need to be evaluated. Also, the initial assembly sequence obtained generally needs to be optimised, filtered, and re-evaluated. (1) Optimise filtering: Using TransRate (http://hibberdlab.com/transrate/) (Smith-Unna et al., 2016) and CD-HIT (http://weizhongli-lab.org/cd-hit/) software (Li and Godzik, 2006); (2) Assembly evaluation: Using BUSCO software (Benchmarking Universal Single-Copy Objects, http://busco.ezlab.org) (Simão et al., 2015).

All transcripts obtained from this transcriptional group sequencing were compared with six databases (NR, Swiss-Prot, Pfam, COG, GO, and KEGG databases), obtaining annotated information in each database and making statistics on each database annotation.

### Sequencing data analysis

The transcripts per million reads (TPM) values of all genes were used to analyse the correlation between each sample (Conesa et al., 2016). To further reveal the molecular events of the biosynthesis of *P. chinensis* triterpenoid saponins, we used DESeq2 (Robles et al., 2012) to analyse the expression levels with |log2FC| ≥ 1 and P_adjust_ < 0.001 (Li et al., 2014; Love et al., 2014; Gao et al., 2020) in at least one of the comparisons, which were considered differential expressed genes (DEGs) for further analysis.

DEGs were analysed for functional enrichment including GO enrichment and KEGG enrichment (Ashburner et al., 2000; Kanehisa and Goto, 2000). Goatools was used for unigene/transcript GO enrichment analysis using Fisher’s exact test. When the adjusted P-value (P_adjust_) < 0.05, the function was significantly enriched. KEGG pathway enrichment analysis of unigenes was carried out. The KEGG pathway was used for unigene enrichment analysis, and the calculation principle was the same as GO functional enrichment analysis. When the corrected P-value (P_adjust_) < 0.05, KEGG pathway was considered to be significantly enriched.

### Terpenoid metabolite analysis

Biological samples were freeze-dried with a vacuum freeze-dryer (Scientz-100F). The freeze-dried sample was crushed using a mixer mill (MM 400, Retsch) with a zirconia bead for 1.5 min at 30 Hz. Dissolve 100 mg of lyophilised powder with 1.2 mL of 70% methanol solution, vortex 30 s every 30 min for six times in total, and place the sample in a refrigerator at 4°C overnight. Following centrifugation at 12,000 rpm for 10 min, the extracts were filtrated (SCAA-104, 0.22 μm pore size; ANPEL, Shanghai, China, http://www.anpel.com.cn/) before UPLC-MS/MS analysis.

The sample extracts were analysed using an UPLC-ESI-MS/MS system (UPLC, SHIMADZU Nexera X2, https://www.shimadzu.com.cn/; MS, Applied Biosystems 4500 Q TRAP, https://www.thermofisher.cn/cn/zh/home/brands/applied-biosystems.html). The analytical conditions were as follows: UPLC: column, Agilent SB-C18 (1.8 µm, 2.1 mm * 100 mm). The mobile phase consisted of solvent A (pure water with 0.1% formic acid) and solvent B (acetonitrile with 0.1% formic acid). Sample measurements were performed with a gradient program that employed the starting conditions of 95% A and 5% B. Within 9 min, a linear gradient of 5% A and 95% B was programmed, and a composition of 5% A and 95% B was kept for 1 min. Subsequently, a composition of 95% A and 5.0% B was adjusted within 1.1 min and kept for 2.9 min. The flow velocity was set as 0.35 mL per minute. The column oven was set to 40°C. The injection volume was 4 μl. The effluent was alternatively connected to an ESI-triple quadrupole-linear ion trap (QTRAP)-MS.

LIT and triple quadrupole (QQQ) scans were acquired on a triple quadrupole-linear ion trap mass spectrometer (Q TRAP), AB4500 Q TRAP UPLC/MS/MS System, equipped with an ESI Turbo Ion-Spray interface, operating in positive and negative ion mode and controlled by Analyst 1.6.3 software (AB Sciex). The ESI source operation parameters were as follows: ion source, turbo spray; source temperature, 550°C; ion spray voltage (IS), 5500 V (positive ion mode)/-4500 V (negative ion mode); ion source gas I (GSI), gas II(GSII), and curtain gas (CUR) were set at 50, 60, and 25.0 psi, respectively; the collision-activated dissociation was high. Instrument tuning and mass calibration were performed with 10 and 100 μmol/L polypropylene glycol solutions in QQQ and LIT modes, respectively. QQQ scans were acquired as multiple reaction monitoring (MRM) experiments with collision gas (nitrogen) set to medium. DP and CE for individual MRM transitions was done with further DP and CE optimisation. A specific set of MRM transitions was monitored for each period according to the metabolites eluted within this period.

Qualitative and quantitative analyses of the metabolites were performed using secondary spectral information based on the public metabolite database and the self-built MWBD database (Wuhan Metware Biotechnology Co., Ltd., China).

Qualitative and quantitative analysis of metabolites in samples were based on local metabolic database and the self-built MWBD database (Wuhan Metware Biotechnology Co., Ltd., China).

According to the secondary spectrum information, the material was qualitatively characterised. Substance characterisation were based on secondary spectral information. Isotopic signals; duplicate signals containing K^+^ ions, Na^+^ ions, and NH4^+^ ions; and repeating signals of fragment ions that are themselves other larger molecular weight species were removed from the analysis. Metabolite quantification was performed using triple-quadrupole mass spectrometry in MRM mode. Mass spectral data were processed using Analyst 1.6.3 software.

Principal component analysis was performed on the samples using multivariate statistical analysis (Chen et al., 2009). Orthogonal partial least squares discriminant analysis (OPLS-DA) models were used to analyse metabolomic data to further demonstrate differences between groups (Thévenot et al., 2015). Based on OPLS-DA results, differentially accumulated metabolites (DAMs) were screened according to fold change ≥2 or fold change ≤0.5 and VIP ≥1. At the same time, the obtained DAMs were submitted to the KEGG website for related pathway analysis.

### qRT-PCR analysis

According to the sequenced transcriptome data, the TPM value of gene expression was greater than 50, and the TPM value between samples was basically the same as the screening standard (Ma Lulin et al., 2019). The relative amounts of these genes were verified using qRT-PCR. The primer sequences were provided by Beijing Dingguo Changsheng Biotechnology Co., Ltd.

The RNA was extracted from different samples following the instructions in the Plant Total RNA Isolation Kit (Forgene Co., Ltd.). The purity and concentration were checked by NanoDrop2000, and the integrity of the total RNA was examined using 1% agarose gel electrophoresis. The RNA was synthesized into cDNA in accordance with RT Easy™ II(With gDNase)(Forgene Co., Ltd) for qPCR. The qRT-PCR reaction was performed using CFX Connect ™ Real-Time System(Bio-Rad) and ChamQ SYBR Color qPCR Master Mix (2X) (Vazyme Biotech Co., Ltd.). The following were used for the qRT-PCR reaction system: cDNA, 0.8 μl; upstream and downstream primers, 0.8 μl; 2X ChamQ SYBR Color qPCR Master Mix 10 μl, and ddH2O, 8.4 μl. The cycle conditions were as follows: pre-denaturation at 95°C for 30 s, followed by cycling for 44 rounds (95°C for 10 s, 72°C for 30 s). The gene expression changes between different sample materials were calculated using the 2^-ΔΔCt^ method, with three biological replicates (Livak and Schmittgen, 2001).

## Results

### Effects of SA on callus growth and accumulation of saponin B4 in *P. chinensis*

The SA is a hormone elicitor that is commonly used to regulate secondary metabolism in medicinal plants. At two key time points of callus suspension growth (18 and 30 days), SA was added to co-culture with callus. The results showed that SA mainly affected the sugar concentration, pH value, electrical conductivity, and saponin B4 content.

**Figure 2:**
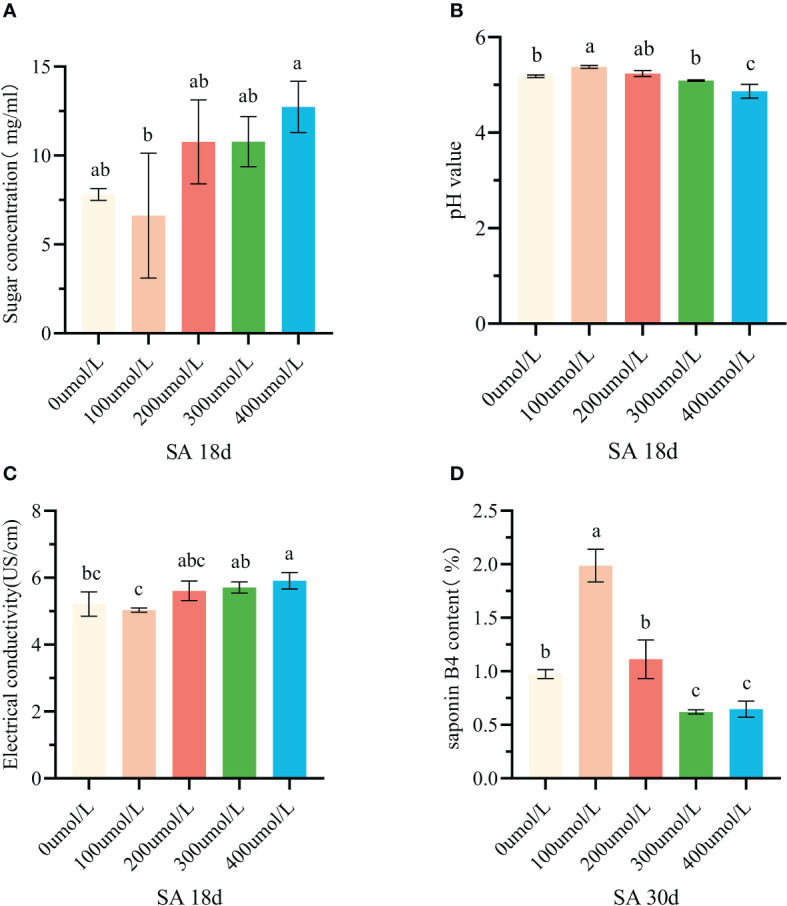
Effects of salicylic acid on callus growth and accumulation of saponin B4 at different stages. **(A)** Change of sugar concentration in culture medium after adding salicylic acid for 18 days; **(B)** Change of pH value in culture medium after adding salicylic acid for 18 days; **(C)** Change of conductivity in culture medium after adding salicylic acid for 18 days; **(D)** Changes of saponin b4 content in callus after adding salicylic acid for 30d of culture. Note: There was no significant difference between the same lowercase English letters (p>0.05). There was significant difference between the different lowercase English letters (p<0.05)

A certain concentration of SA was added into the system of suspension callus which had cultured for 18 days. The sugar concentration of the culture medium firstly decreased and then increased gradually (**Figure 2A**). Adding 100μmol/L SA, callus consumed the most nutrients. The pH value of culture medium firstly increased and then decreased, and the pH value under treatment of 400μmol/L SA was significantly lower than that of the control group (**Figure 2B**). The conductivity of the culture medium was the lowest under100μmol/L SA, but there was no significant difference between it and the control group. When the concentration of SA was further increased, the conductivity gradually increased. The conductivity under treatment of 400μmol/L SA was the highest, which was significantly higher than that of the control group (**Table 1**, **Figure 2C**). The index component of Pulsatilla saponin B4 changed significantly after adding SA, when the callus was cultured for 30 days. Under the treatment of 100μmol/L SA, the B4 content increased significantly and reached a peak of 1.99% (**Figure 2D**). When the SA concentration was further increased, the B4 content decreased, and the 300μmol/L treatment decreased to the lowest level.

**Table 1:** The effect of SA elicitor on the growth of callus of *P. chinensis*.

	treatment (umol/L)	18d			SA 30d
No.		sugar concentration (mg/ml)	pH value	conductivity (Us/cm)	B4 content(%)
1	0	7.81±0.19ab	5.18±0.02b	5.21±0.21bc	0.97±0.02b
2	100	6.62±2.03b	5.38±0.02a	5.03±0.04c	1.99±0.09a
3	200	10.77±1.37ab	5.24±0.04ab	5.61±0.17abc	1.11±0.10b
4	300	10.78±0.82ab	5.09±0.01b	5.71±0.10ab	0.62±0.01c
5	400	12.74±0.84a	4.87±0.08c	5.91±0.14a	0.65±0.04c

### *De novo* assembly and functional annotation

Based on the analysis results in the previous section, 100 μmol/L SA was to be added after 30 days of callus suspension culture. *P. chinensis* callus was co-cultured with SA for 0, 1, 2, and 3 days respectively for transcriptome sequencing analysis, and each treatment was replicated three times. A total of 316.9 million clean reads were assembled into 140,794 unigenes, with an average length of 745.54 bp, GC percentage of 40.88%, N50 of 1242 bp, and E90N50 of 2637 bp. Amongst these unigenes, the maximum length was 17,628 bp, and the minimum length was 201 bp (**Table 2**). The mapped ratio was greater than 80% (**Table 3**). The assembly results are good for subsequent analysis.

**Table 2:** Transcriptome de novo assembly results.

Type	Unigene
Total number	140794
Total base	1.05E+08
Largest length (bp)	17628
Smallest length (bp)	201
Average length (bp)	745.54
N50 length (bp)	1242
E90N50 length (bp)	2637
Fragment mapped percent(%)	67.508
GC percent (%)	40.88
TransRate score	0.24777
BUSCO score	C:75.9%[S:73.1%;D:2.8%]

**Table 3:** Comparison of sequencing data and assembly results.

Sample	Clean reads	Mapped reads	Mapped ratio
SA_CK1	26140352	21471343	82.14%
SA_CK2	29605049	24264901	81.96%
SA_CK3	30036229	24784073	82.51%
SA_1D1	28424342	23305726	81.99%
SA_1D2	26425489	21746351	82.29%
SA_1D3	24508701	20040122	81.77%
SA_2D1	26056717	21579779	82.82%
SA_2D2	24675713	20404738	82.69%
SA_2D3	25574990	20912542	81.77%
SA_3D1	24673920	20306062	82.30%
SA_3D2	23526063	19465369	82.74%
SA_3D3	27294170	22374277	81.97%

**Figure 3:**
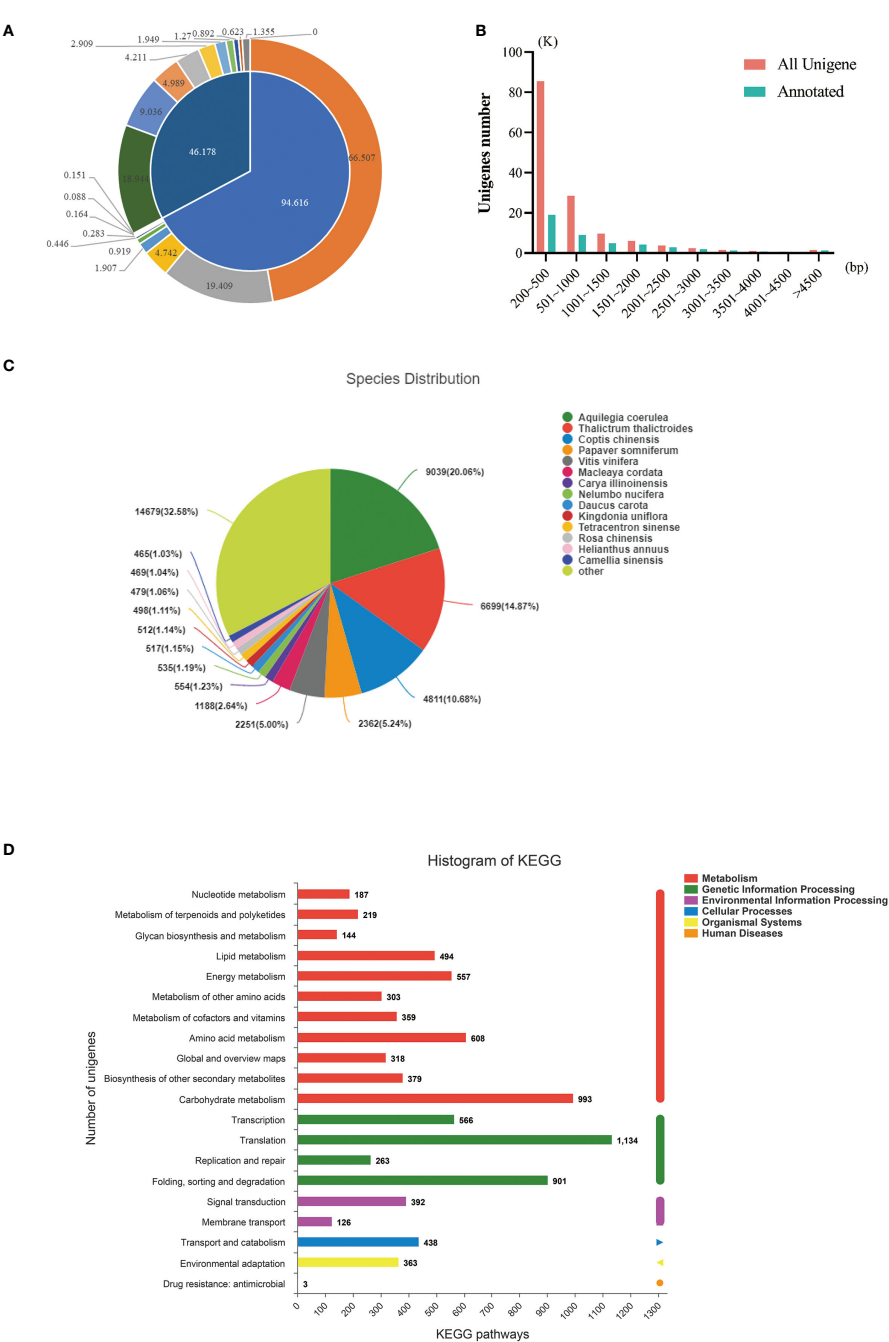
De novo assembly and functional annotation of transcriptome sequencing. **(A)** The overview of sequencing data annotation; **(B)** Length distribution statistics of annotated and unannotated unigenes; **(C)** NR annotation result; **(D)** KEGG annotation results. Note:K means its number is 1000;bp means base pair.

Then, the results obtained from the assembly were compared with six major databases. Amongst them, 46,178 unigenes (32.80%) were annotated, and 94,616 (67.2%) were not annotated (**Table 4**, **Figure 3A**). The length distribution statistics of both annotated and unannotated unigenes were mainly concentrated in 200-500 bp, which were 85,451 (61%) and 18,944 (41.02%), respectively (**Figure 3B**). The most annotated data were obtained from the NR database, with 45,058 (32%) unigenes being annotated (**Table 5**, **Supplementary Figure 1**). The KEGG database had the least annotation information, and 12,543 (8.91%) unigenes were annotated (**Table 5**, **Supplementary Figure 1**). A total of 8332 unigenes were annotated in all databases (**Supplementary Figure 1**).

**Table 4:** Sequence length distribution and annotation statistics.

Type	Number (K)	Length	Number (K)
Unannotated	94.616	200~500	66.507
		501~1000	19.409
		1001~1500	4.742
		1501~2000	1.907
		2001~2500	0.919
		2501~3000	0.446
		3001~3500	0.283
		3501~4000	0.164
		4001~4500	0.088
		>4500	0.151
Annotated	46.178	200~500	18.944
		501~1000	9.036
		1001~1500	4.989
		1501~2000	4.211
		2001~2500	2.909
		2501~3000	1.949
		3001~3500	1.27
		3501~4000	0.892
		4001~4500	0.623
		>4500	1.355

**Table 5:** Annotation information statistics.

Database	All_Unigene number(percent)
GO	36059(0.2561)
KEGG	12543(0.0891)
COG	29496(0.2095)
NR	45058(0.32)
Swiss-Prot	24698(0.1754)
Pfam	25890(0.1839)
Total_anno	46178(0.328)
Total	140794(1)

In the NR database, amongst the species with high matching degree of similar bands, *Aquilegia coerulea* accounted for the highest proportion (16.87%), followed by *Arabidopsis* (13.72%), *Coptis chinensis* (9.83%), and *Papaver somniferum* (5.97%) (**Figure 3C**). A total of 12,543 (8.91%) gene annotations were found in the KEGG database, and KEGG metabolic pathways were divided into six categories: metabolism, genetic information processing, environmental information processing, cellular processes, organic systems, and human diseases. Amongst them, metabolism contains the most genes, with a total of 4561 genes, and carbohydrate metabolism was the main metabolic pathway (**Figure 3D**).

### DEGs identification and enrichment analyses

**Figure 4:**
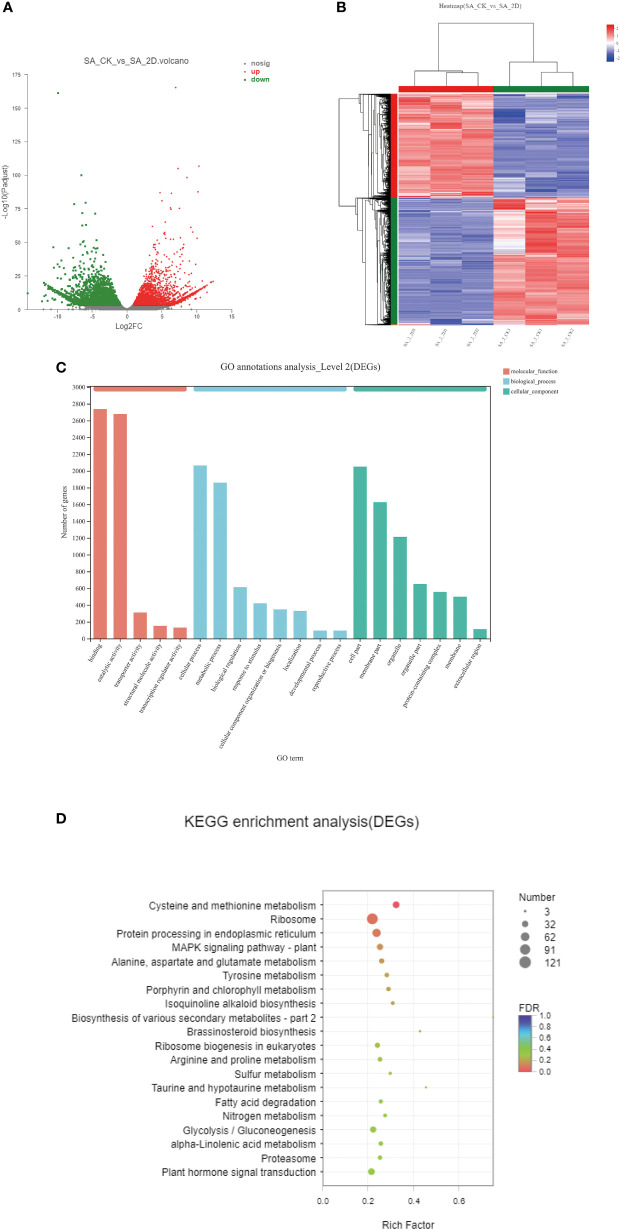
DEGs enrichment analyses. **(A)** DEGs volcano map between the SA_CK and SA_2D; **(B)** The hierarchical clustering of DEGs between the SA_CK and SA_2D; **(C)** GO enrichment analysis of DEGs; **(D)** KEGG enrichment analysis of DEGs.

The TPM values were calculated for each unigene expression. The significant DEGs were selected by setting |log2FC| ≥ 2 and P_adjust_ ≤ 0.001 as thresholds. Thus, a total of 9558 significant DEGs were observed in six comparison groups (**Table 6**). Amongst them, the largest number of significant DEGs (3635 upregulated and 4404 downregulated unigenes) was detected between the SA_CK and SA_2D groups (**Table 6**, **Figure 4A**).

**Table 6:** Statistics on the number of DEGs between samples under SA stress treatment.

Diff_group	Total DEGs	Up	Down
SA_CK_vs_SA_1D	7760	3560	4200
SA_1D_vs_SA_2D	56	10	46
SA_2D_vs_SA_3D	17	8	9
SA_1D_vs_SA_3D	73	24	49
SA_CK_vs_SA_2D	8039	3635	4404
SA_CK_vs_SA_3D	5292	2323	2969

The hierarchical clustering of DEGs between the SA_CK and SA_2D groups is shown in **Figure 4B**. Red and blue indicate high and low expression levels, respectively. The expression patterns of the SA_CK and SA_2D libraries revealed that SA_ CK1, SA_ CK2, and SA_ CK3 and SA_2D1, SA_2D2, and SA_2D3 were classified into the same cluster. To better understand the biological functions of DEGs, the significant DEGs from six comparison groups were analysed using GO and KEGG method. The DEGs were mainly enriched into three GO categories: molecular function, cellular component, and biological process (**Figure 4C**). Amongst them, the DEGs in the molecular function category are mainly annotated under the entries of binding and catalytic activity; the DEGs in the biological process category were mainly annotated under the entries of cellular process and metabolic process; the DEGs in the cellular component category were mainly annotated under the entries of cell part and membrane part.

The GO enrichment directed acyclic graph was also used to illustrate the GO structure of DEGs (**Supplementary Figure 2**). The closer the colour is to red, the more significantly enriched this GO term is. The line between the GO terms represents the relationship between the two GOs. For KEGG analysis, the significant DEGs were mainly enriched in the “metabolic pathways” and “genetic information processing “ pathways (**Figure 4D**).

### Gene expression analysis of terpenoid biosynthesis pathway

A total of 9558 DEGs were obtained by comparing six different groups. KEGG pathway enrichment analysis was performed on these DEGs, and 122 pathways were enriched (**Supplementary Table 3**). For terpenoid synthesis-related genes, 13, 5, 4, 11, 4, 18, and 12 DEGs were enriched in terpenoid backbone biosynthesis; monoterpenoid biosynthesis; diterpenoid biosynthesis; ubiquinone and other terpenoid-quinone biosynthesis pathways; lysine biosynthesis; phenylalanine, tyrosine and tryptophan biosynthesis; and phenylalanine metabolism, respectively.

**Figure 5:**
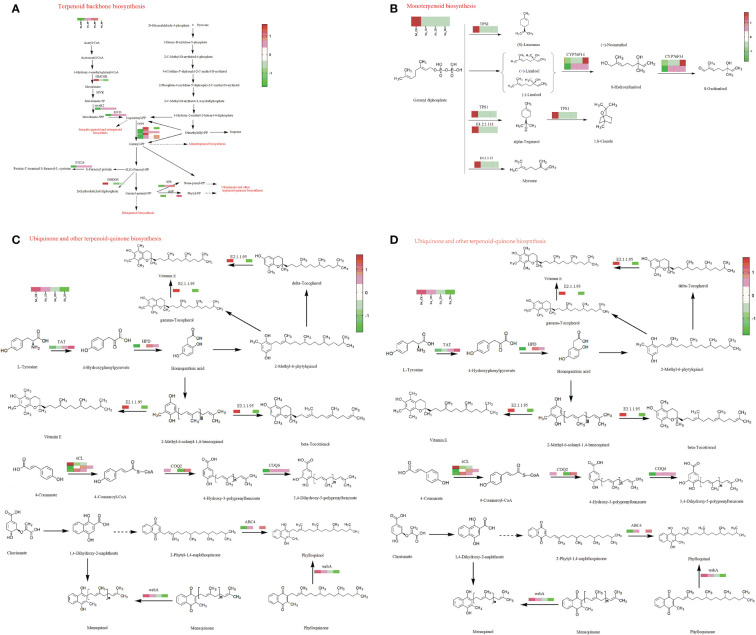
DEGs involved in Terpenoid Biosynthesis Pathways; **(A)**The DEGs enriched in the terpenoid backbone synthesis pathway; **(B)** The DEGs enriched in the monoterpene synthesis pathway; **(C)** The DEGs enriched in the diterpene synthesis pathway; **(D)** The DEGs enriched in the ubiquinone and other terpenoid quinone synthesis pathway.

The DEGs enriched in the terpenoid backbone synthesis pathway mainly encode HMCGR, mvaK2, MVD, DHDDS, GGPS, chlP, and SPS (**Supplementary Table 4**, **Figure 5A**). The DEGs enriched in the monoterpene biosynthesis pathway mainly encode E4.2.3.15, E4.2.3.111, CYP76F14and TPS1 (**Supplementary Table 4**, **Figure 5B**). The DEGs enriched in the diterpene biosynthesis pathway mainly encode CYP701, KAO, GA3ox, and GA2ox (**Supplementary Table 4**, **Figure 5C**). The DEGs enriched in ubiquinone and other terpenoid quinone biosynthesis pathways mainly encode 4CL, TAT, HPD, E2.1.1.95, COQ2, COQ6, ABC4, and wrbA (**Supplementary Table 4**, **Figure 5D**). The DEGs enriched in the lysine biosynthesis pathway mainly encode lysC, dapA, dapB, and lysA (**Supplementary Figure 3** and **Table 4**). The DEGs enriched in the phenylalanine, tyrosine and tryptophan biosynthesis pathway mainly encode trpA, trpB, aroDE, aroK, aroL, trpE, ADT, PDT, GOT1, TAT, and TYRAAT (**Supplementary Figure 4** and **Table 4**). The DEGs enriched in the phenylalanine metabolic pathway mainly encode GOT1, TAT, HPD, and E3.5.1.4 (**Supplementary Figure 5** and **Table 4**).

### Terpenoid DAM analysis

**Figure 6:**
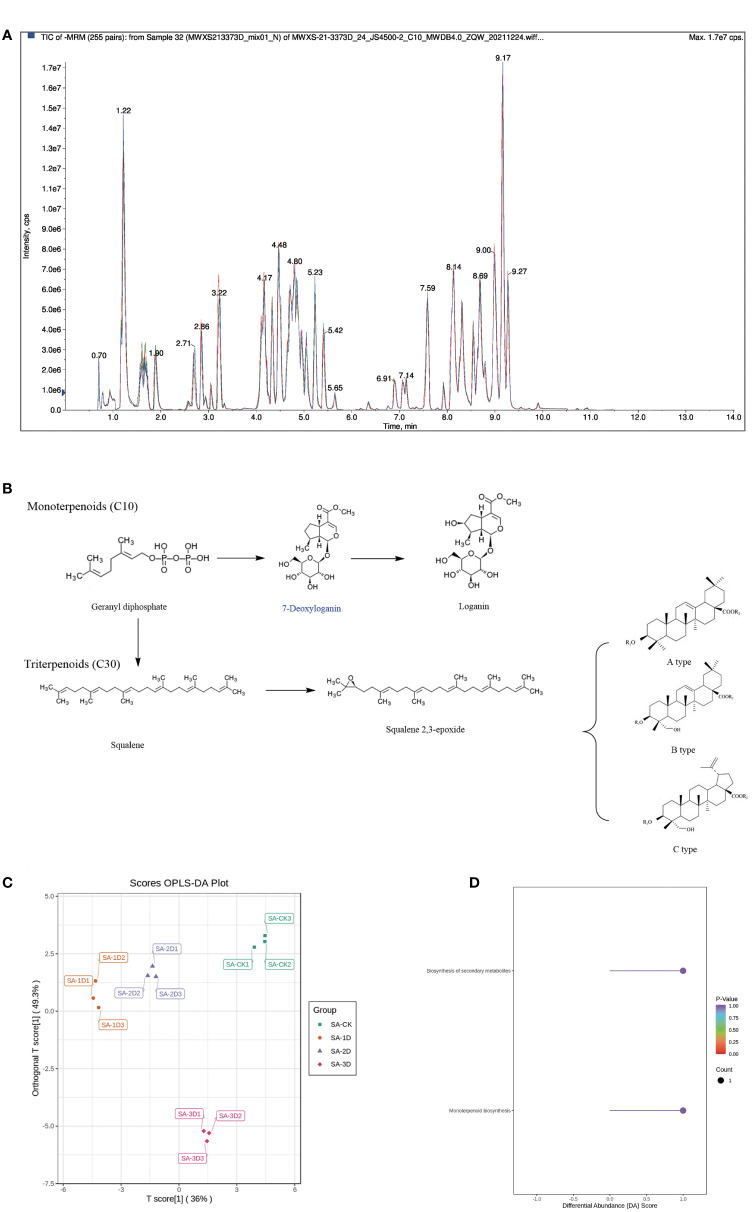
Terpenoid DAMs Analysis. **(A)** QC sample mass spectrometry detection TIC overlay; **(B)** DAMs involved in triterpene synthesis pathway; **(C)** Metabolic analysis using OPLS-DA; **(D)** KEGG enrichment analysis of DAMs.

Before analysing DAMs, we obtained the total ion current (TIC) of QC samples. The results showed that the curves of TIC detected by metabolites had high overlap, that is, the retention time and peak intensity were consistent, indicating that the signal stability of mass spectrometry was good when the same sample was detected at different times (**Figure 6A**). In this study, OPLS-DA was performed on the samples to discriminate the variation between and within each sample group of SA_CK, SA_1D, SA_2D, and SA_3D. Amongst them, the contribution rates of PC1 and PC2 are 36.0% and 49.3%, respectively. These two principal components can basically reflect the main characteristic information of the test sample (**Figure 6C**). The four groups showed an obvious separation on the two-dimensional graph, indicating that the data of each sample were credible, and there were obvious differences amongst the samples (**Figure 6C**).

In this study, we analysed the resulting metabolic profiles using targeted metabolomics techniques and identified a total of 31 terpenoids in four groups (**Table 7**). To further understand the metabolic changes in *P. chinensis* callus under SA stress, univariate and multivariate statistical analyses were performed to determine the DAMs in each treatment group compared with the control group, with a threshold of VIP ≥ 1 and a fold change ≥ 2 or ≤ 0.5. Amongst them, 22 terpenoid DAMs were identified in SA-CK_vs_SA-1D, 22 terpenoid DAMs in SA-CK_vs_SA-2D, and 23 terpenoid DAMs in SA-CK_vs_SA-3D. The DAMs of the above three groups were analysed, and a total of 31 DAMs were found.

**Table 7:** Summary of identification of terpenoids in P. chinensis callus under SA treatment.

No.	Formula	Compounds	Class
1	C_16_H_24_O_9_	7-Deoxyloganic acid	Monoterpenoids
2	C_16_H_24_O_10_	Loganic acid	Monoterpenoids
3	C_30_H_48_O_3_	Ursolic acid	Triterpene
4	C_30_H_46_O_4_	Hederagonic acid (23-Hydroxy-3-oxoolean-12-en-28-oic acid)	Triterpene
5	C_41_H_66_O_12_	α-Hederin	Triterpene Saponin
6	C_41_H_66_O_12_	Pulchinenoside A;Anemoside A3	Triterpene Saponin
7	C_41_H_66_O_12_	Hederagenin-3-O-α-L-rhamnopyranosyl-(1→2)-α-L-arabinopyranoside	Triterpene Saponin
8	C_41_H_66_O_13_	Hederagenin-3-O-β-D-glucopyranosyl-(1→4)-α-L-arabinopyranoside	Triterpene Saponin
9	C42H68O14	3β,23-Dihydroxylup-20(29)-en-28-oic acid-28-O-β-D-glucopyranosyl-(1→6)-β-D-glucopyranoside	Triterpene Saponin
10	C_47_H_76_O_16_	Pulchinenoside B10	Triterpene Saponin
11	C_47_H_76_O_16_	Pulchinenoside B7	Triterpene Saponin
12	C_47_H_76_O_17_	3-O-α-L-rhamnopyranosyl-(1→2)-[ β-D-glucopyranosyl-(1→4)]-α-L-arabinopyranosyl-3β,23-dihydroxylup-Δ20(29)-en-28-oic acid*	Triterpene Saponin
13	C_47_H_76_O_17_	Hederagenin-3-O-β-D-glucopyranosyl-(1→3)-α-L-rhamnopyranosyl-(1→2)-α-L-arabinopyranoside*	Triterpene Saponin
14	C_47_H_76_O_17_	Hederagenin-3-O-α-L-rhamnopyranosyl-(1→2)-[(β-D-glucopyranosyl-(1→4)]-α-L-arabinopyranoside	Triterpene Saponin
15	C_47_H_76_O_17_	Pulsatilla saponin D*	Triterpene Saponin
16	C_47_H_76_O_18_	3-O-α-L-arabinopyranosyl-Hederagenin-28-O-β-D-glucopyranosyl-(1→6)-β-D-glucopyranoside	Triterpene Saponin
17	C_48_H_78_O_18_	Hederagenin-28-O-β-D-rhamnopyranosyl-(1→4)-α-L-glucopyranosyl-(1→6)-α-L-glucopyranoside*	Triterpene Saponin
18	C_48_H_78_O_18_	3β,23-Dihydroxylup-20(29)-en-28-oic acid-28-O-α-L-rhamnopyranosyl-(1→4)-β-D-glucopyranosyl-(1→4)-β-D-glucopyranoside*	Triterpene Saponin
19	C_48_H_78_O_18_	Hederagenin-28-O-α-L-rhamnopyranosyl-(1→4)-β-D-glucopyranosyl-(1→6)-β-D-glucopyranoside	Triterpene Saponin
20	C_53_H_86_O_21_	Pulchinenoside E2	Triterpene Saponin
21	C_53_H_86_O_22_	Pulchinenoside B*	Triterpene Saponin
22	C_53_H_86_O_22_	Pulchinenoside E1;Macranthoside B*	Triterpene Saponin
23	C_53_H_86_O_22_	Hederacoside D	Triterpene Saponin
24	C_54_H_88_O_23_	Cirenshenoside S	Triterpene Saponin
25	C_59_H_96_O_25_	Oleanolic acid-3-O-α-L-rhamnopyranosyl-(1→6)-β-D-glucopyranosyl-(1→4)-β-D-glucopyranosyl-(1→3)-α-L-rhamnopyranosyl-(1→2)-α-L-arabinopyranoside	Triterpene Saponin
26	C_59_H_96_O_26_	Hederacoside C	Triterpene Saponin
27	C_59_H_96_O_26_	Pulchinenoside B4;Pulchinenoside C;Anemoside B4	Triterpene Saponin
28	C_59_H_96_O_26_	3-O-β-D-glucopyranosyl-(1→3)-α-L-rhamnopyranosyl-(1→2)-α-L-arabinopyranosyl-Oleanolic acid-O-β-D-glucopyranosyl-(1→6)-β-D-glucopyranoside	Triterpene Saponin
29	C_59_H_96_O_27_	3-O-β-D-glucopyranosyl-(1→4)-α-L-arabinopyranosyl-Hederagenin-28-O-α-L-rhamnopyranosyl-(1→4)-β-D-glucopyranosyl-(1→6)-β-D-glucopyranoside	Triterpene Saponin
30	C_59_H_96_O_27_	Macranthoidin A*	Triterpene Saponin
31	C_59_H_96_O_27_	3-O-β-D-glucopyranosyl-(1→4)-[α-L-rhamnopyranosyl-(1→2)]-α-L-arabinopyranosyl-Hederagenin-28-O-β-D-glucopyranosyl-(1→6)-β-D-glucopyranoside*	Triterpene Saponin

Note:“*”limited by the detection principle of mass spectrometry, these substances have the same molecular weight and ionic fragment information, and they are isomers, which cannot be distinguished in the mass spectrometry results.

On this basis, the literature was reviewed to analyse the chemical constituents in the original plants of *P. chinensis* that have been reported (Xu Li-Ke; Xiujuan, 2010; Ding Xiujuan et al., 2010; Zhan, 2012; Huijun, 2012; Pu Qiaoli et al., 2021). Compared with the results of this experiment, a total of 21 DAMs have been reported. Deoxyloganic acid belongs to monoterpenoids, and the other 20 DAMs belong to triterpene saponin(**Figure 6B**).

Amongst the remaining 20 triterpenoid saponins, 4 compounds including pulchinenoside B7 belong to A-type triterpenoid saponins; 12 compounds such as hederagenin-3-O-α-L-rhamnopyranosyl-(1→2)-α-L-arabinopyranoside belong to B-type triterpenoid saponins; 4 compounds including anemoside B4 belong to C-type triterpenoid saponins (Pu Qiaoli et al., 2021) (**Figure 6B**).

The DAMs in the three differential groups were annotated using the KEGG database, and pathway enrichment analysis was performed. The significantly enriched pathways were screened with P < 0.01 as the threshold, and two highly enriched pathways were obtained, namely, the secondary metabolic biosynthesis pathway and the monoterpene biosynthesis pathway (**Figure 6D**).

Expression profile analysis by qRT-PCR

Six DEGs were randomly selected for quantitative real-time PCR (qRT-PCR) to validate their expression levels during the process of SA resistance in *P. chinensis* (Bunge) Regel. The RT-qPCR results found that the transcriptional levels of the tested genes were in a correlated trend with the respective abundance estimated by RNA-seq (**Supplementary Figure 6**), suggesting relative rationality and accuracy of the transcriptome analysis in this study. There are four DEGs (TRINITY_DN22653_c0_g2, TRINITY_DN2292_c0_g1, TRINITY_DN7817_c0_g2, TRINITY_DN5169_c0_g1) enriched in the Pyruvate metabolism pathway (map00620). Pyruvate is an important substance in the terpenoid skeleton synthesis pathway, which is closely related to the synthesis and metabolism of terpenoids (Ma, 2011; Qian Guan et al., 2011; Boronat and Rodríguez-Concepción, 2015).

## Discussion

The formation and accumulation of secondary metabolites in medicinal plants are regulated by a variety of factors. In recent years, environmental factors, hormone regulation, and the expression and regulation of functional genes had often been reported (Martins et al., 2018; Ibrahim et al., 2019; Dugasa et al., 2020). The early results showed that the growth period of suspension culture callus was about 40 days: the slow growth period was 0-9 days; the exponential growth period was 12-24 days; the platform period is 27-39d; the decline period was 39 days after culture (**Supplementary Figure 7**). After consulting the literature and combining the early experimental results, we finally chose two key time points of callus suspension culture: 18 days (middle exponential growth stage) and 30 days (early platform growth stage) for experiments (Wenjing Huang et al., 2012; Jinting Li et al., 2016; Pan, 2019). In the present study, the callus of *P. chinensis* was selected as the material, and a certain concentration of SA hormone elicitor was added at two key time points to determine the growth of callus and the content of *Pulsatilla* saponin B4, an index component of the pharmacopoeia. On this basis, transcriptomic and metabolomic techniques were used to analyse the transcriptional and metabolic changes of *P. chinensis* callus under the regulation of SA. Functional genes affecting secondary metabolism of *P. chinensis* and metabolic pathways with significant enrichment were analysed. Thus, a foundation will be built for the excavation of functional genes related to the synthesis of saponins from *P. chinensis* and the analysis of metabolic pathways.

### SA regulates the growth and accumulation of secondary metabolites in *P. chinensis* callus

In this paper, fresh weight, pH value, electrical conductivity, sugar concentration and saponin B4 content were selected to compare the changes of callus growth of *P. chinensis* under salicylic acid stress. Among them, fresh weight is generally used to analyse the proliferation efficiency of callus (Wenjing Huang et al., 2012; Xiaozhen, 2020). The pH value and conductivity of the culture medium are used to measure the cell membrane permeability of the callus and judge the health status of the callus (Wenjing Huang et al., 2012; Zhang et al., 2016). However, the sugar concentration is to reflect the carbon source absorbed by callus (Jinting Li et al., 2016). Saponin B4 has been used to evaluate the quality and pharmacological activity of *P. chinensis* (Commission, 2020; Li et al., 2020). Salicylic acid, as a hormone inducer, has been widely proved to have certain effects on the accumulation of secondary metabolites of medicinal plants (Guo, 2016; Yongbo Duan et al., 2017; Ruixiang Lei et al., 2021). After 30 days of callus suspension culture, 100μmol/L of SA was added. In this paper, after 30 days of suspension culture of callus, 100 μmol/L salicylic acid was added, and after 2 days of co culture, the accumulation of Pulsatilla saponin B4 in callus was significantly higher than that in control group (1.99 ± 0.09)%. Consistent with the studies of Zhang Yuanyuan (Zhang Yuanyuan et al., 2019) and Zhang Jingyi (Juan-E, 2015), the SA hormone elicitor has a certain regulatory effect on the synthesis and accumulation of secondary metabolites in medicinal plants.

### Determination of gene expression changes in *P. chinensis* callus induced by SA through transcriptome sequencing technology

About 100μmol/L of SA was added to the *P. chinensis* callus proliferation culture system and co-cultured for 0, 1, 2, and 3 days. Then, it was taken out for transcriptome sequencing research. In NR species annotation, *Aquilegia* has the highest matching degree, but its proportion is only 16.87%. There are very few studies related to the gene function of *P. chinensis*, and some only focused on DNA barcoding and chloroplast genome sequencing (Yang Wei et al., 2011; Qiujie, 2019; Tingting, 2021). The research on the functional gene of *P. chinensis* needs to be enriched.

By setting |log2FC| ≥ 2 and P_adjust_ ≤ 0.001, a total of 9558 significant DEGs were obtained. Compared with the control group, the number of DEGs obtained by SA_2D sequencing was the highest (up to 8039). These DEGs were significantly enriched in 122 pathways, including multiple pathways for terpenoid synthesis and metabolism, especially terpenoid backbone biosynthesis, monoterpenoid biosynthesis, diterpenoid biosynthesis, and ubiquinone and other terpenoid-quinone biosynthesis pathways. Some terpenoid synthesis precursor metabolism pathways were also significantly enriched, such as lysine biosynthesis; phenylalanine, tyrosine and tryptophan biosynthesis; and phenylalanine metabolism.

In-depth analysis of DEGs in terpenoid metabolism-related pathways revealed that multiple genes were significantly enriched. Amongst them, MVD, GGPS, and SPS genes in the terpenoid backbone synthesis pathway have also been reported in *Arabidopsis* (Cordier et al., 1999; Okada et al., 2000; Hirooka et al., 2005). The TPS gene was isolated from *Santalum album* (Santalaceae) and expressed heterologously in *Escherichia coli*, producing a variety of sesquiterpenoids, indicating that the TPS gene is involved in the biosynthesis of sandalwood essential oil (Jones et al., 2008). In addition, the TPS-Cin gene is expressed in *Arabidopsis* root, directing the biosynthesis of volatile monoterpenes (Chen et al., 2004). The ADT gene plays a role in the synthesis of phenylalanine, a precursor required for the synthesis of terpenes (Cho et al., 2007). In addition, some homologous sequences were found in the biosynthetic pathway such as GGPS, CYP76F14, 4CL, GOT1, etc (**Supplementary Figure 8**).

### Metabolomic technique to determine the metabolic changes of terpenoids in *P. chinensis* callus induced by SA

Terpenoids are the main active components of *P. chinensis*, and a variety of terpenoid monomer components have been proved to have significant pharmacological activities (Huihui Song et al., 2021; Liu et al., 2021; Ye et al., 2021). Metabolomics technology was used to analyse the effect of SA elicitors on the metabolic changes of terpenoids in *Pulsatilla* callus. A TIC map of the samples was drawn, and the samples were analysed by OPLS-DA. There are large differences between groups, and different treatments have obvious changes in samples.

Four differential groups were analysed, and a total of 31 DAMs were obtained. Amongst them, 21 components have been reported in the chemical composition analysis of *Pulsatilla*. Except for deoxyloganic acid, which is a monoterpenoid, the rest are triterpenoids. SA elicitor significantly affected the metabolic changes of triterpenoids in *P. chinensis* callus.

In general, this study used plant tissue culture technology, transcriptome sequencing technology, and metabolome technology to elucidate that SA elicitors can affect the metabolic changes of terpenoids in *P. chinensis* callus.

The results showed that SA could promote the accumulation of *Pulsatilla* saponin B4. A number of synthetic pathways related to *P. chinensis* biosynthesis were significantly enriched, and some functional genes were excavated. Metabolomic analysis also showed that triterpenoid metabolism is affected by SA elicitors. In addition, from the analysis of transcriptome sequencing results, the number of genes that can be annotated is relatively small. Approximately 16.87% species were annotated in NR, and only 8.91% were annotated in the KEGG database. The number of related studies on plant genes of *P. chinensis* is relatively small.

On the other hand, from the analysis of transcriptome data, multiple synthetic pathways of terpenoid components in *P. chinensis* were significantly enriched, and a certain number of DEGs were obtained. From metabolomic data analysis, multiple terpenoid component DAMs were identified. However, almost no DEGs related to the accumulation of terpenoids were found, and the joint analysis of DEGs and DAMs was not carried out in this study (Mao et al., 2021; Bai et al., 2021). Overall, these results show that SA elicitor can regulate the metabolism of terpenoids in *P. chinensis* callus and obtain some functional genes related to terpenoid biosynthesis, which provide a basis for analysing the genetic regulation of *P. chinensis* terpenoids.

## Data availability statement

The datasets presented in this study can be found in online repositories. The names of the repository and accession number can be found below: NCBI SRA BioProject, accession no: PRJNA895484.

## Author contributions

SZ and HX conceived the study, participated in its design, coordinated, drafted and revised the manuscript. YD participated in study design and coordination, performed the experimental measurements, processed the experimental data, interpreted the data, and drafted and revised the manuscript. QQ and YC performed the experimental measurements and helped in sampling. ZM helped collect experimental samples. GZ and XW contributed to the design and helped draft the manuscript. All authors contributed to the article and approved the submitted version.

## Supplementary material

Supplementary Figure 1 Annotation results of six databases through UpSetR.

Supplementary Figure 2 The GO enrichment directed acyclic graph.

Supplementary Figure 3 The DEGs enriched in the lysine biosynthesis pathway.

Supplementary Figure 4 The DEGs enriched in the phenylalanine, tyrosine and tryptophan biosynthesis pathway.

Supplementary Figure 5 The DEGs enriched in the phenylalanine metabolic pathway.

Supplementary Figure 6 qRT-PCR confirmation of DEGs.

Supplementary Figure 7 Growth curve of callus suspension culture.

Supplementary Figure 8 Expression level of homologous sequences involved terpenoid biosynthesis pathway.

Supplementary Table 3 DEGs of KEGG pathway enrichment analysis.
