# Supplementary figures and images for: Integrated transcriptomic and metabolomic analyses revealed the molecular mechanism of terpenoid formation for salicylic acid resistance in Pulsatilla chinensis callus
Source: Front Plant Sci. 2023 Jan 6;13:1054317. doi: 10.3389/fpls.2022.1054317 (PMC9854134; doi:10.3389/fpls.2022.1054317)

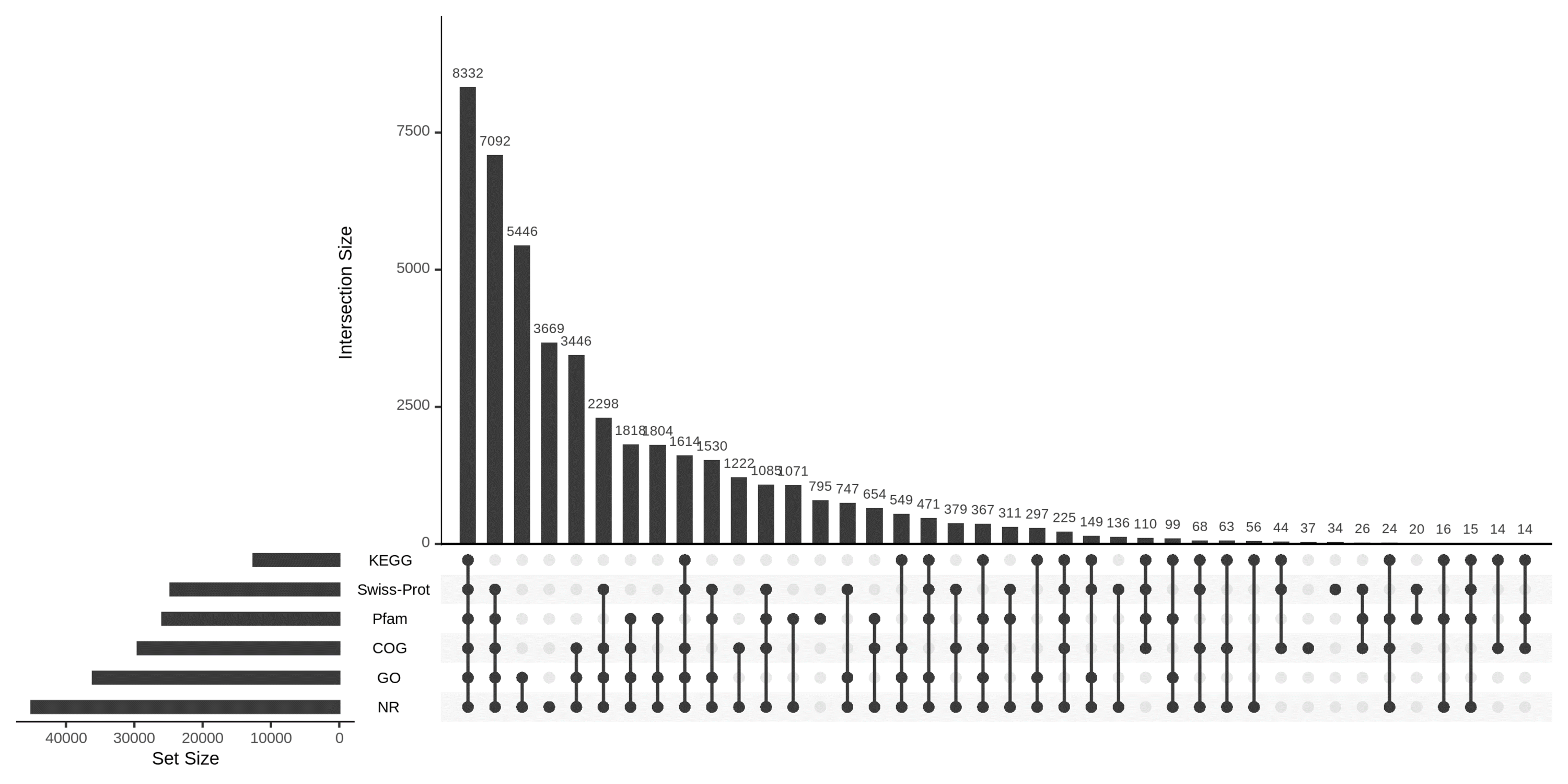

Supplement: Supplementary Figure 1 — Annotation results of six databases through UpSetR. [file Image_1.tif]

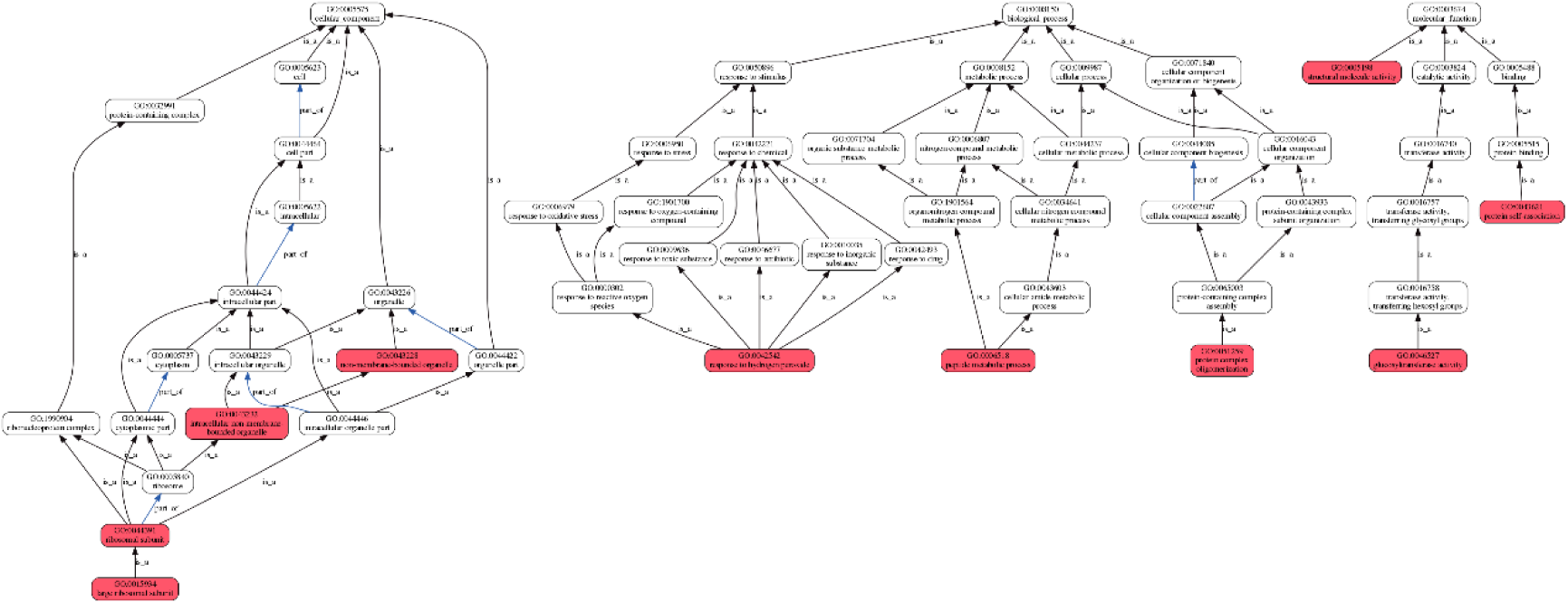

Supplement: Supplementary Figure 2 — The GO enrichment directed acyclic graph. [file Image_2.tif]

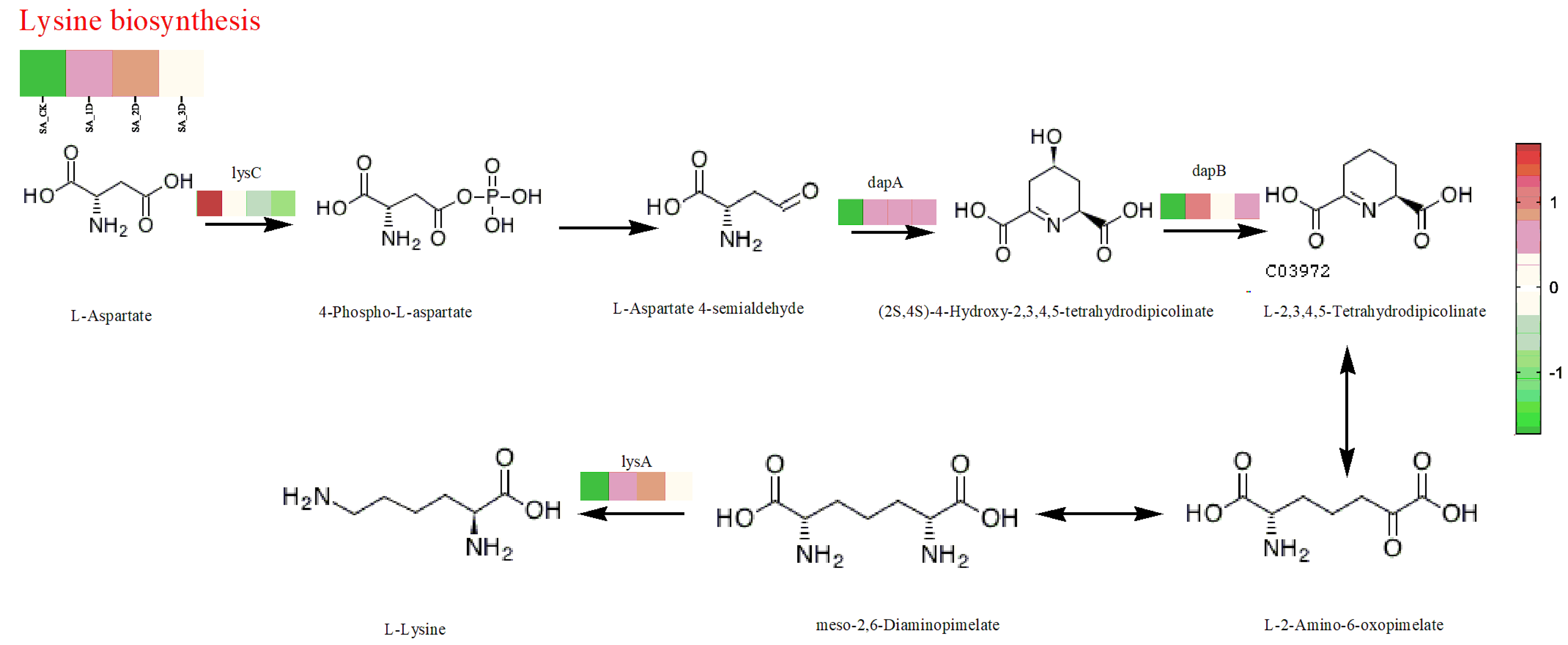

Supplement: Supplementary Figure 3 — The DEGs enriched in the lysine biosynthesis pathway. [file Image_3.tif]

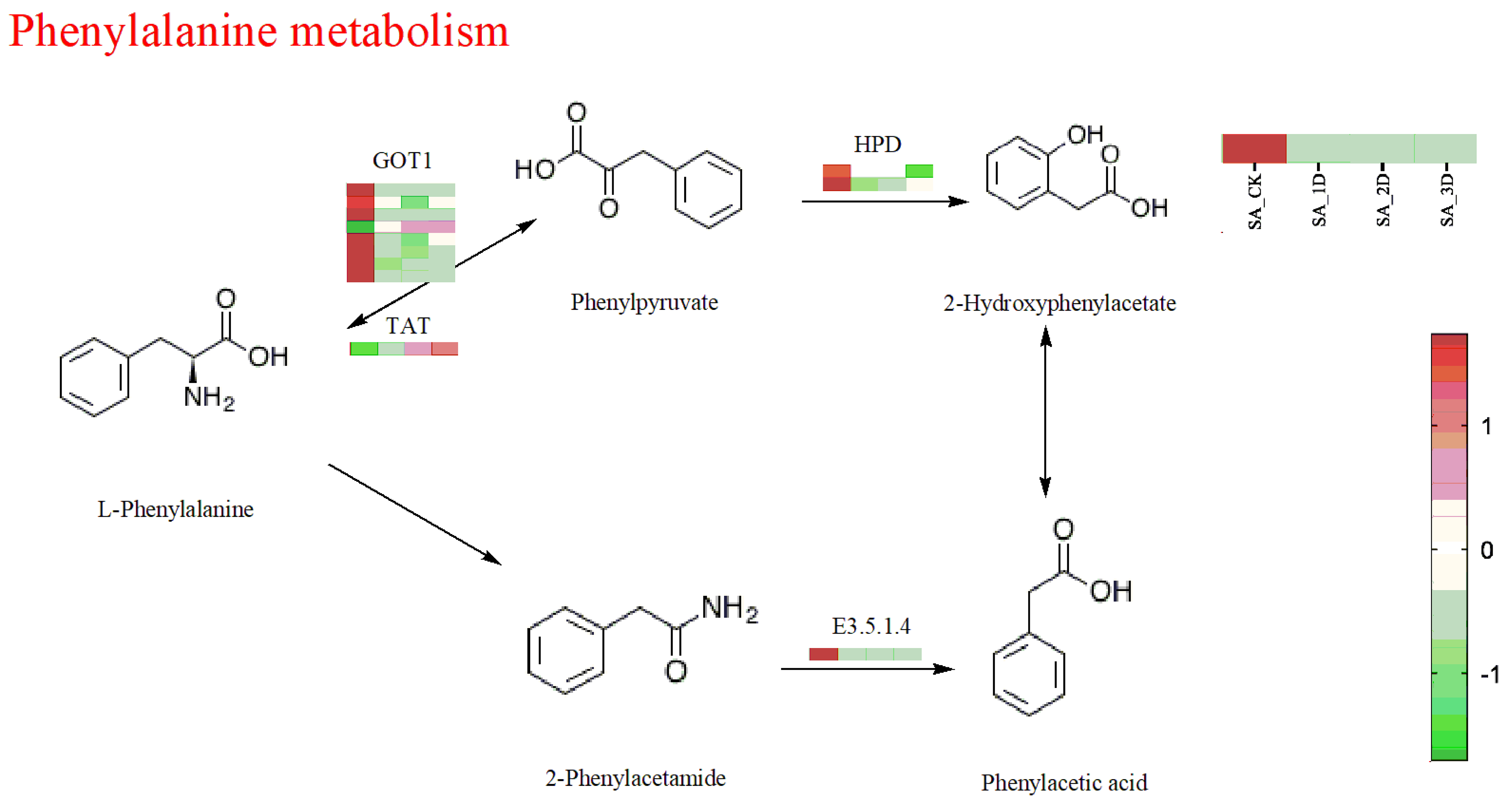

Supplement: Supplementary Figure 4 — The DEGs enriched in the phenylalanine, tyrosine and tryptophan biosynthesis pathway. [file Image_4.tif]

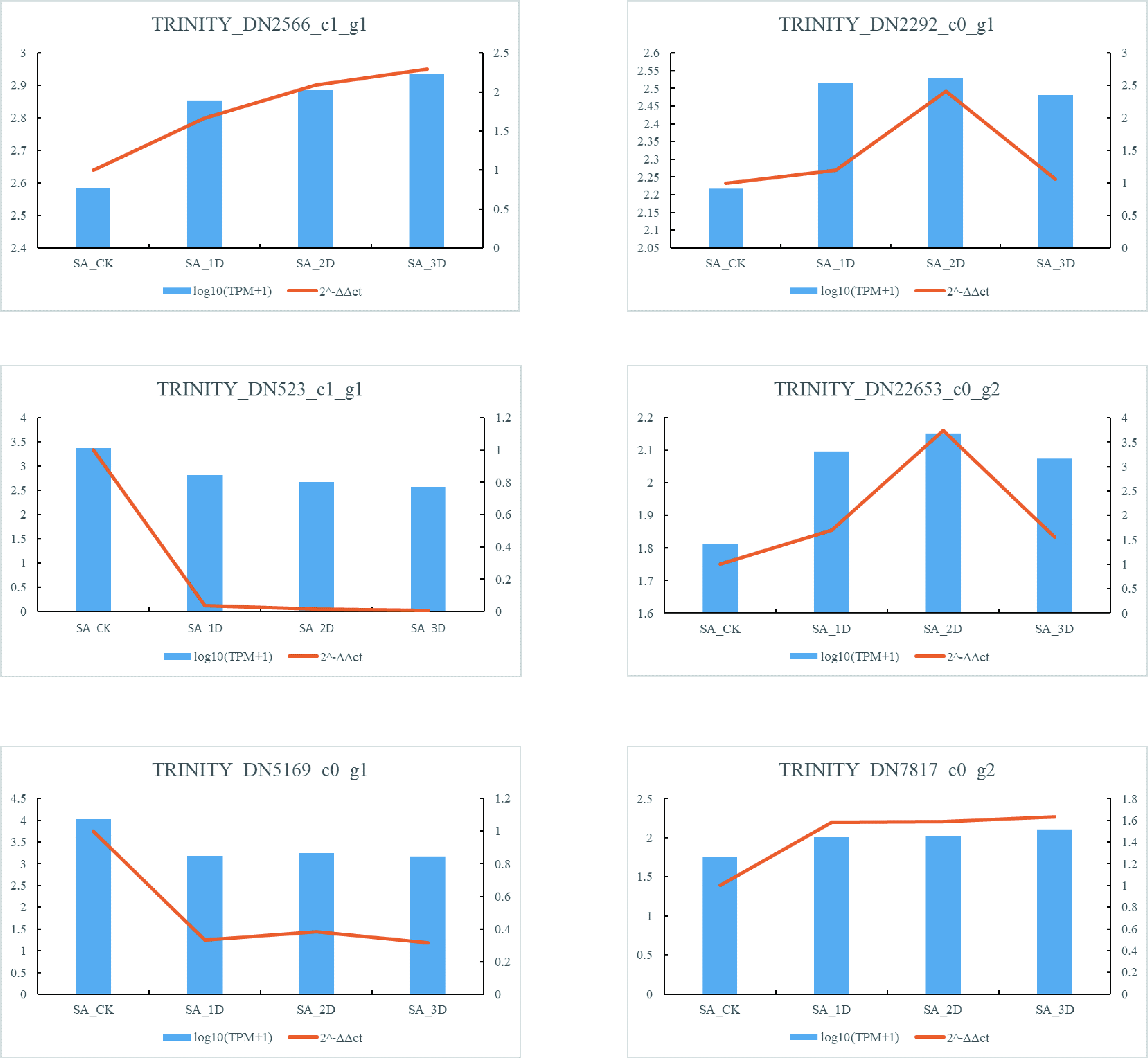

Supplement: Supplementary Figure 5 — The DEGs enriched in the phenylalanine metabolic pathway. [file Image_5.jpeg]

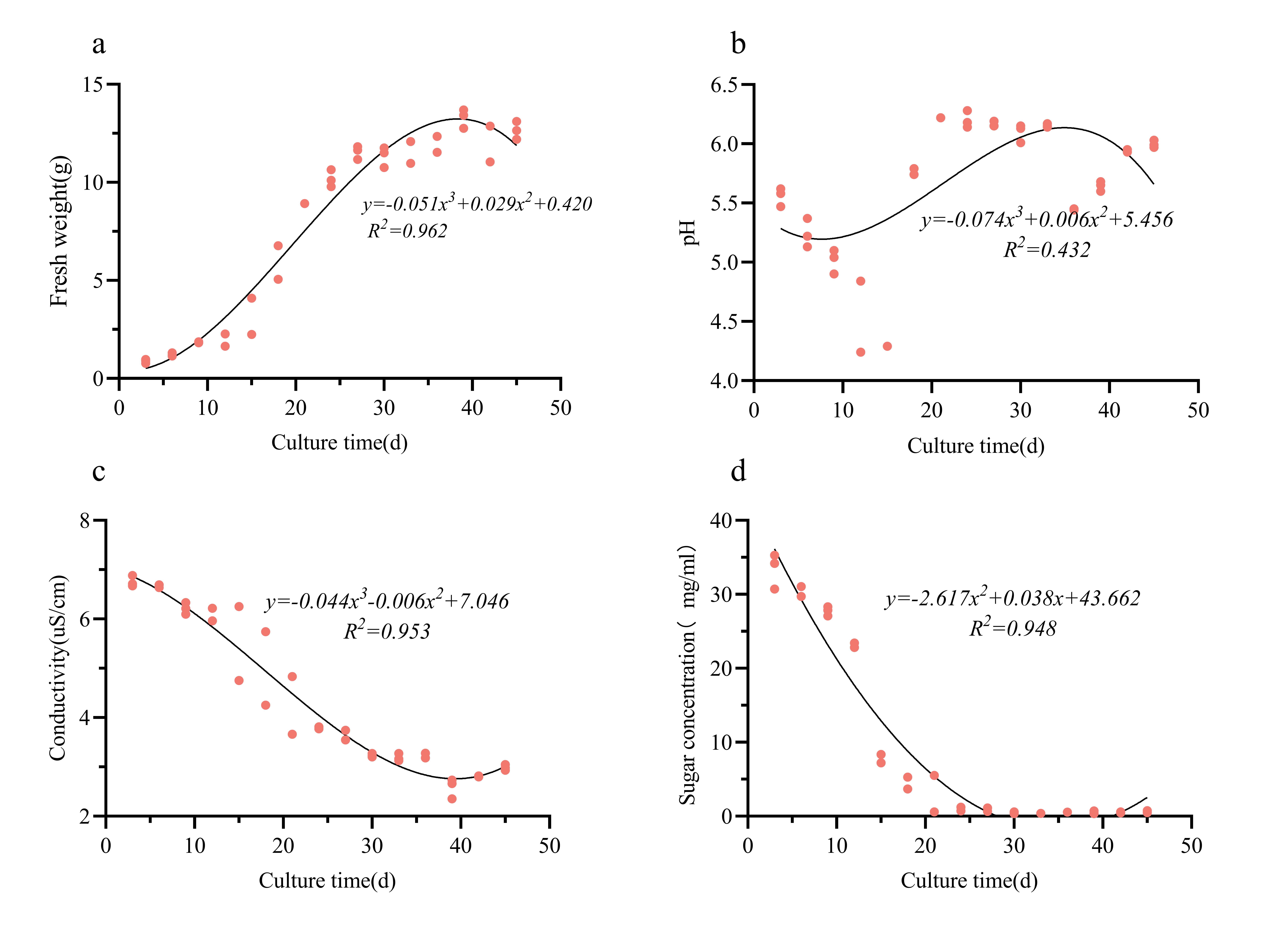

Supplement: Supplementary Figure 6 — qRT-PCR confirmation of DEGs. [file Image_6.tif]

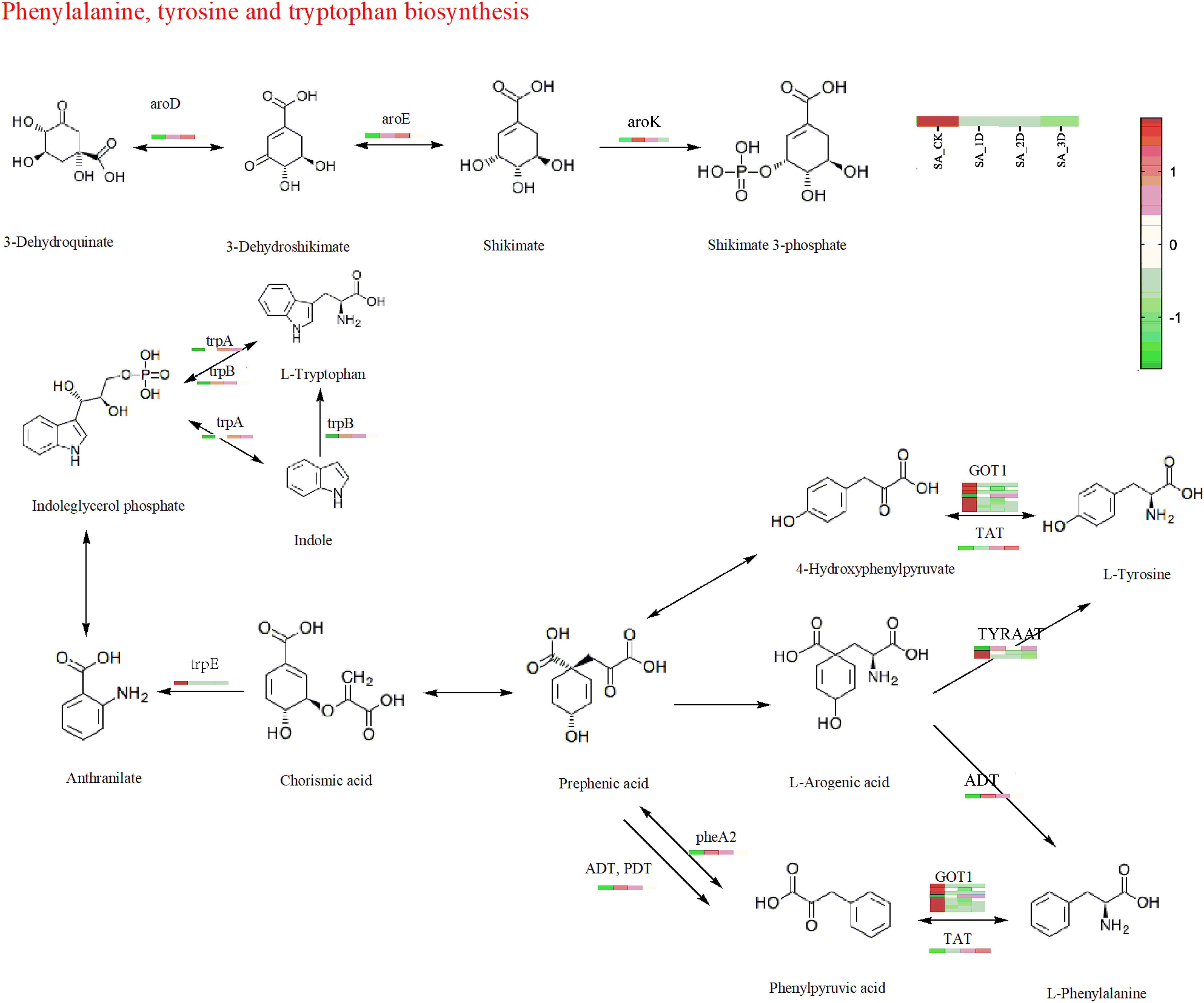

Supplement: Supplementary Figure 8 — Expression level of homologous sequences involved terpenoid biosynthesis pathway. [file Image_8.jpeg]
