# Supplementary material for: Integrated transcriptomic and metabolomic analyses revealed the molecular mechanism of terpenoid formation for salicylic acid resistance in Pulsatilla chinensis callus
Source: Front Plant Sci. 2023 Jan 6;13:1054317. doi: 10.3389/fpls.2022.1054317 (PMC9854134; doi:10.3389/fpls.2022.1054317)
Supplement: Supplementary file 10 [file Table_1.docx]

Supplementary Table 1 Sample RNA quality test results

| No. | sample name | concentration (ng/μl) | total amount (μg) | OD260/280 | OD260/230 | RIN |
| --- | --- | --- | --- | --- | --- | --- |
| 1 | SA_CK1 | 763.30 | 26.72 | 2.17 | 1.28 | 9.80 |
| 2 | SA_CK2 | 1127.30 | 39.46 | 2.19 | 1.52 | 9.90 |
| 3 | SA_CK3 | 1618.40 | 56.64 | 2.18 | 1.88 | 10.00 |
| 4 | SA_1D1 | 1148.60 | 40.20 | 2.22 | 2.21 | 10.00 |
| 5 | SA_1D2 | 631.70 | 22.11 | 2.20 | 2.18 | 10.00 |
| 6 | SA_1D3 | 783.80 | 27.43 | 2.22 | 2.17 | 9.70 |
| 7 | SA_2D1 | 962.50 | 33.69 | 2.22 | 2.21 | 10.00 |
| 8 | SA_2D2 | 543.20 | 19.01 | 2.00 | 1.94 | 9.20 |
| 9 | SA_2D3 | 922.20 | 32.28 | 2.25 | 2.17 | 8.80 |
| 10 | SA_3D1 | 671.30 | 23.50 | 2.22 | 2.08 | 10.00 |
| 11 | SA_3D2 | 1092.00 | 38.22 | 2.20 | 2.22 | 10.00 |
| 12 | SA_3D3 | 493.70 | 17.28 | 2.09 | 1.97 | 9.90 |
