# Supplementary material for: Integrated transcriptomic and metabolomic analyses revealed the molecular mechanism of terpenoid formation for salicylic acid resistance in Pulsatilla chinensis callus
Source: Front Plant Sci. 2023 Jan 6;13:1054317. doi: 10.3389/fpls.2022.1054317 (PMC9854134; doi:10.3389/fpls.2022.1054317)
Supplement: Supplementary file 11 [file Table_2.docx]

Supplementary Table 2 Quality control analysis of sequencing data

| Sample | Clean reads | Clean bases | Error rate(%) | Q20(%) | Q30(%) | GC content(%) |
| --- | --- | --- | --- | --- | --- | --- |
| SA_CK1 | 52280704 | 7.59E+09 | 0.0252 | 97.93 | 93.93 | 44.58 |
| SA _CK2 | 59210098 | 8.61E+09 | 0.0254 | 97.87 | 93.79 | 44.70 |
| SA _CK3 | 60072458 | 8.68E+09 | 0.0252 | 97.90 | 93.94 | 44.91 |
| SA_1D1 | 56848684 | 8.31E+09 | 0.0252 | 97.93 | 93.93 | 44.61 |
| SA_1D2 | 52850978 | 7.74E+09 | 0.0253 | 97.89 | 93.82 | 44.60 |
| SA_1D3 | 49017402 | 7.21E+09 | 0.0258 | 97.73 | 93.41 | 44.59 |
| SA_2D1 | 52113434 | 7.57E+09 | 0.0256 | 97.78 | 93.56 | 44.63 |
| SA_2D2 | 49351426 | 7.20E+09 | 0.0253 | 97.90 | 93.87 | 44.68 |
| SA_2D3 | 51149980 | 7.49E+09 | 0.027 | 97.29 | 92.28 | 44.71 |
| SA_3D1 | 49347840 | 7.17E+09 | 0.0257 | 97.73 | 93.45 | 44.68 |
| SA_3D2 | 47052126 | 6.91E+09 | 0.0255 | 97.81 | 93.64 | 44.77 |
| SA_3D3 | 54588340 | 7.94E+09 | 0.0258 | 97.67 | 93.45 | 44.41 |
