# Supplementary material for: Integrated transcriptomic and metabolomic analyses revealed the molecular mechanism of terpenoid formation for salicylic acid resistance in Pulsatilla chinensis callus
Source: Front Plant Sci. 2023 Jan 6;13:1054317. doi: 10.3389/fpls.2022.1054317 (PMC9854134; doi:10.3389/fpls.2022.1054317)
Supplement: Supplementary file 12 [file Table_4.docx]

Supplementary Table 4 DEGs information involved in terpenoid biosynthetic pathway

| Pathway id | KO name | Name | Gene_id |  |
| --- | --- | --- | --- | --- |
| map00900(Terpenoid backbone biosynthesis) | HMGCR | Hydroxy methyl glutaryl-CoA reductase | TRINITY_DN903_c0_g1 |  |
|  | MVK | mevalonate kinase | TRINITY_DN8197_c0_g1 |  |
|  | mvaK2 | phosphomevalonate kinase | TRINITY_DN5342_c0_g1 |  |
|  | MVD | diphosphomevalonate decarboxylase | TRINITY_DN47673_c0_g5 |  |
|  | GGPS | geranylgeranyl diphosphate synthase, type III | TRINITY_DN4026_c0_g1;  TRINITY_DN6495_c1_g2; TRINITY_DN56375_c0_g2; TRINITY_DN88285_c1_g1; TRINITY_DN6077_c0_g1; TRINITY_DN435_c0_g1 |  |
|  |  |  |  |  |
|  |  |  |  |  |
|  |  |  |  |  |
|  |  |  |  |  |
|  |  |  |  |  |
|  | STE24 | STE24 endopeptidase | TRINITY_DN9020_c0_g1 |  |
|  | DHDDS, RER2, SRT1 | ditrans, polycis-polyprenyl diphosphate synthase | TRINITY_DN11091_c0_g3 |  |
|  | chlP, bchP | geranylgeranyl diphosphate/geranylgeranyl-bacteriochlorophyllide a reductase | TRINITY_DN32271_c0_g1 |  |
|  | SPS, sds | all-trans-nonaprenyl-diphosphate synthase | TRINITY_DN12620_c0_g1 |  |
| map00902(Monoterpenoid biosynthesis) | E4.2.3.15 | myrcene/ocimene synthase | TRINITY_DN7236_c0_g1 |  |
|  | E4.2.3.111 | (-)-alpha-terpineol synthase | TRINITY_DN31169_c0_g1; TRINITY_DN7236_c0_g1 |  |
|  |  |  |  |  |
|  | TPS1 | (+)-alpha-terpineol/(4S)-limonene synthase | TRINITY_DN8934_c0_g2 |  |
|  | CYP76F14 | (E)-8-carboxylinalool synthase | TRINITY_DN20716_c0_g5; TRINITY_DN15516_c1_g1 |  |
|  |  |  |  |  |
| map00904(Diterpenoid biosynthesis) | GA3, CYP701 | ent-kaurene oxidase | TRINITY_DN12658_c2_g1 |  |
|  | KAO | ent-kaurenoic acid monooxygenase | TRINITY_DN33825_c0_g1 |  |
|  | GA2ox | gibberellin 2beta-dioxygenase | TRINITY_DN8909_c0_g1 |  |
|  | GA3ox | gibberellin 3beta-dioxygenase | TRINITY_DN1711_c1_g1 |  |
| map00130(Ubiquinone and other terpenoid-quinone biosynthesis) | HPD, hppD | 4-hydroxyphenylpyruvate dioxygenase | TRINITY_DN2760_c1_g1 |  |
|  | 4CL | 4-coumarate--CoA ligase | TRINITY_DN38419_c0_g1; TRINITY_DN9317_c0_g1; TRINITY_DN2852_c0_g2 |  |
|  |  |  |  |  |
|  |  |  |  |  |
|  | TAT | tyrosine aminotransferase | TRINITY_DN5951_c0_g1 |  |
|  | E2.1.1.95 | tocopherol O-methyltransferase | TRINITY_DN966_c0_g1 |  |
|  | COQ2 | 4-hydroxybenzoate polyprenyltransferase | TRINITY_DN643_c0_g1 |  |
|  | COQ6 | ubiquinone biosynthesis monooxygenase Coq6 | TRINITY_DN3683_c1_g1 |  |
|  | wrbA | NAD(P)H dehydrogenase (quinone) | TRINITY_DN6178_c0_g1 |  |
|  | ABC4, menA | 2-carboxy-1,4-naphthoquinone phytyltransferase | TRINITY_DN10881_c0_g1 |  |
| map00400(Phenylalanine, tyrosine and tryptophan biosynthesis) | aroDE, DHQ-SDH | 3-dehydroquinate dehydratase / shikimate dehydrogenase | TRINITY_DN7537_c0_g2 |  |
|  | trpA | tryptophan synthase alpha chain | TRINITY_DN468_c0_g2 |  |
|  | trpB | tryptophan synthase beta chain | TRINITY_DN6772_c0_g1 |  |
|  | aroK, aroL | shikimate kinase | TRINITY_DN412_c0_g1 |  |
|  | trpE | anthranilate synthase component I | TRINITY_DN46084_c0_g2 |  |
|  | ADT, PDT | arogenate/prephenate dehydratase | TRINITY_DN7654_c1_g1 |  |
|  | GOT1 | aspartate aminotransferase, cytoplasmic | TRINITY_DN89024_c0_g1; TRINITY_DN21788_c0_g1; TRINITY_DN2711_c1_g3; TRINITY_DN2711_c1_g1; TRINITY_DN41213_c0_g1; TRINITY_DN15941_c0_g1; TRINITY_DN789_c1_g1; TRINITY_DN13133_c0_g1 |  |
|  |  |  |  |  |
|  |  |  |  |  |
|  |  |  |  |  |
|  |  |  |  |  |
|  | TAT | tyrosine aminotransferase | TRINITY_DN5951_c0_g1 |  |
|  | TYRAAT | arogenate dehydrogenase (NADP+), plant | TRINITY_DN19675_c0_g2; TRINITY_DN19426_c0_g1; TRINITY_DN19288_c0_g1 |  |
|  |  |  |  |  |
|  |  |  |  |  |
| map00300(Lysine biosynthesis) | lysC | aspartate kinase | TRINITY_DN1360_c0_g4 |  |
|  | dapA | 4-hydroxy-tetrahydrodipicolinate synthase | TRINITY_DN7872_c0_g4 |  |
|  | dapB | 4-hydroxy-tetrahydrodipicolinate reductase | TRINITY_DN4024_c0_g1 |  |
|  | lysA | diaminopimelate decarboxylase | TRINITY_DN24107_c0_g1 |  |
| map00360(Phenylalanine metabolism） | GOT1 | aspartate aminotransferase, cytoplasmic | TRINITY_DN89024_c0_g1; TRINITY_DN21788_c0_g1; TRINITY_DN2711_c1_g3; TRINITY_DN2711_c1_g1; TRINITY_DN41213_c0_g1; TRINITY_DN15941_c0_g1; TRINITY_DN789_c1_g1; TRINITY_DN13133_c0_g1 |  |
|  |  |  |  |  |
|  |  |  |  |  |
|  |  |  |  |  |
|  |  |  |  |  |
|  |  |  |  |  |
|  |  |  |  |  |
|  |  |  |  |  |
|  | TAT | tyrosine aminotransferase | TRINITY_DN5951_c0_g1 |  |
|  | HPD, hppD | 4-hydroxyphenylpyruvate dioxygenase | TRINITY_DN2760_c1_g1 |  |
|  | E3.5.1.4, amiE | amidase | TRINITY_DN23430_c0_g3 |  |
